# Buffalo Academy of Medicine

**Published:** 1901-12

**Authors:** F. W. McGuire

**Affiliations:** Secretary


### SOCIETY PROCEEDINGS.

### Buffalo Academy of Medicine.

REPORTED BY F. W. McGUIRE, M. D., Secretary.

THE stated meeting of the Buffalo Academy of Medicine was held at the Academy rooms, Public Library Building, Monday, November 18, 1901. The president, Dr. CHARLES G. STOCKTON, called the meeting to order at 9 p.m.

The minutes of the last stated meeting were read and approved.

The council recommended the election of Drs. Bernard F. Dennis, of Niagara Falls, and William R. Campbell, of Niagara Falls, for nonresident membership. They also recommended the election of Drs. James A. Gardner, Earl P. Lothrop, F. A. Drake, Ulysses B. Stein, Jacob Goldberg, Jacob M. Kraus, H. A. Kendal, George S. Staniland, Herman K. DeGroat, Francis M. O'Gorman, Julien A. Riester, Clark E. Ernest, George Roberts, William Irving Thornton, Arthur McCarthy, George A. Sloan, A. McNiel Blair, James J. Mooney, J. F. Whitwell, and Bert C. Johnson for resident membership.

They were separately balloted for and elected Fellows of the Academy.

The Hartwig prize was presented to Dr. Irving P. Lyon by Dr. Stockton in his usual able manner. In accepting it Dr. Lyon made appropriate remarks.

Dr. JEWETT moved a vote of thanks to Dr. Grover Wende for the large number of members he had secured for the Academy.

The section of ophthalmology provided the following program, The operation for extraction of cataract, as originally devised by Dariel, A. A. Hubbell.

Dr. B. H. GROVE presented three cases: (1) Iron in the iris; (2) iron in the lens; (3) paresis of the motor oculi of the left eye following a blow.

Dr. A. A. HUBBELL presented an interesting case of exophthalmos of the left eye.

Dr. LUCIEN HOWE explained the necessity of the uniformity of the test type.

Attendance, 32; adjourned at 10.45 p.m.

